# Comparison of causes of stillbirth and child deaths as determined by verbal autopsy and minimally invasive tissue sampling

**DOI:** 10.1371/journal.pgph.0003065

**Published:** 2024-07-29

**Authors:** Nega Assefa, Anthony Scott, Lola Madrid, Merga Dheresa, Gezahegn Mengesha, Shabir Mahdi, Sana Mahtab, Ziyaad Dangor, Nellie Myburgh, Lesego Kamogelo Mothibi, Samba O. Sow, Karen L. Kotloff, Milagritos D. Tapia, Uma U. Onwuchekwa, Mahamane Djiteye, Rosauro Varo, Inacio Mandomando, Ariel Nhacolo, Charfudin Sacoor, Elisio Xerinda, Ikechukwu Ogbuanu, Solomon Samura, Babatunde Duduyemi, Alim Swaray-Deen, Abdulai Bah, Shams El Arifeen, Emily S. Gurley, Mohammed Zahid Hossain, Afruna Rahman, Atique Iqbal Chowdhury, Bassat Quique, Portia Mutevedzi, Solveig A. Cunningham, Dianna Blau, Cyndy Whitney

**Affiliations:** 1 College of Health and Medical Sciences, Haramaya University, Harar, Ethiopia; 2 London School of Hygiene and Tropical Medicine, London, United Kingdom; 3 South African Medical Research Council Vaccines and Infectious Diseases Analytics Research Unit, University of the Witwatersrand, Johannesburg, South Africa; 4 Centre pour le Développement des Vaccins), Ministère de la Santé, Bamako, Mali; 5 Department of Pediatrics, Center for Vaccine Development and Global Health, University of Maryland School of Medicine, Baltimore, Maryland, United States of America; 6 ISGlobal, Hospital Clínic, Universitat de Barcelona, Barcelona, Spain; 7 Centro de Investigação em Saúde de Manhiça, Maputo, Mozambique; 8 Instituto Nacional de Saude, Ministerio de Saude, Maputo, Mozambique; 9 Crown Agents, Freetown, Sierra Leone; 10 World hope international, Makeni, Sierra Leone; 11 University of Sierra Leone Teaching Hospitals Complex, Sierra Leone; 12 University of Ghana Medical School, Accra, Ghana; 13 FOCUS 10000, Freetown, Sierra Leone; 14 Maternal and Child Health Division, International Centre for Diarrhoeal Disease Research Bangladesh, Dhaka, Bangladesh; 15 Bloomberg School of Public Health, Johns Hopkins University, Baltimore, Maryland, United States of America; 16 International Centre for Diarrhoeal Disease Research Bangladesh, Dhaka, Bangladesh; 17 Program for Emerging Infections, Infectious Disease Division, International Centre for Diarrhoeal Disease Research Bangladesh b, Dhaka, Bangladesh; 18 Global Health Institute, Emory University, Atlanta, Georgia, United States of America; 19 Global Health Center, Centers for Disease Control and Prevention, Atlanta, Georgia, United States of America; Makerere University, UGANDA

## Abstract

In resource-limited settings where vital registration and medical death certificates are unavailable or incomplete, verbal autopsy (VA) is often used to attribute causes of death (CoD) and prioritize resource allocation and interventions. We aimed to determine the CoD concordance between InterVA and CHAMPS’s method. The causes of death (CoDs) of children <5 were determined by two methods using data from seven low- and middle-income countries (LMICs) enrolled in the Child Health and Mortality Prevention Surveillance (CHAMPS) network. The first CoD method was from the DeCoDe panel using data from Minimally Invasive Tissue Sampling (MITS), whereas the second method used Verbal Autopsy (VA), which utilizes the InterVA software. This analysis evaluated the agreement between the two using Lin’s concordance correlation coefficient. The overall concordance of InterVA4 and DeCoDe in assigning causes of death across surveillance sites, age groups, and causes of death was poor (0.75 with 95% CI: 0.73–0.76) and lacked precision. We found substantial differences in agreement by surveillance site, with Mali showing the lowest and Mozambique and Ethiopia the highest concordance. The InterVA4 assigned CoD agrees poorly in assigning causes of death for U5s and stillbirths. Because VA methods are relatively easy to implement, such systems could be more useful if algorithms were improved to more accurately reflect causes of death, for example, by calibrating algorithms to information from programs that used detailed diagnostic testing to improve the accuracy of COD determination.

## Introduction

Most low and middle-income countries (LMIC) with high child mortality lack adequate systematic mortality surveillance [1]. For example, death registration coverage varies from nearly 100% in the WHO European region to less than 10% in the WHO African region [2]. In LMICs, deaths are often not attended by health professionals, not medically certified, not recorded in a timely way, and, even when recorded, the information is stored inappropriately [3]. LMICs also do not have the infrastructure or resources to establish and maintain data systems that conclusively identify causes of death in their populations [4]. Not having appropriate legislation or health policies on data systems compounds these challenges, leading to ineffective formulation and implementation of interventions to reduce mortality at the population level [5, 6].

One relatively simple method to identify causes of death (CoDs) is through a verbal autopsy (VA) [7–10]. To conduct a VA, workers who are trained interviewed family members or caregivers of the deceased using a structured questionnaire; they also solicit a qualitative narrative of the circumstances surrounding death [11]. Causes of death (CoDs) can then be generated from the structured questionnaire. These can be decided by physician coding, but, because this is time-consuming and expensive and may be seen as inefficient, especially in resource-limited settings, probabilistic analytic algorithms have been developed and are freely available online. This publicly available CoDs generating software is widely used as a public health tool for mortality estimation and identifying population-level CoDs in resource-limited settings [12, 13].

While the VA method has many benefits, it also has weaknesses [14, 15]. One challenge relates to the quality of the data that can be collected via VAs. The method can produce conflicting and unreliable CoD results because it relies on the quality and accuracy of information provided by family members, who typically lack clinical training. The community’s sociocultural norms, as well as the informants’ recall biases, can affect the responses. The VA forms do not collect information on known or pre-existing medical conditions determined based on diagnostic testing, as the families respondents might not have access to past clinical information [16–18]. There are also challenges in the use of the data to generate cause of death results. The presence of multiple VA algorithms and the tool’s inherent limitations in accurately diagnosing pre-existing or new medical problems make it challenging to assign the CoD for conditions with complex cause-of-death pathways or highly non-specific signs and symptoms [19, 20]. In addition, the VA does not generate the complete mortality pathways, such as the immediate or morbid pathways, but only determines the underlying CoDs with various probabilistic scores, which may not adequately capture complicated medical histories. For stillbirths, the VA only describes the body’s condition, which has been shown to not accurately reflect the cause of death among stillbirths [21, 22].

The Child Health and Mortality Prevention Surveillance (CHAMPS) is a collaborative network in sub-Saharan Africa and South Asia that uses additional approaches, including Minimally Invasive Tissue Sampling (MITS), clinical data, histopathological and microbiological findings, together with the VA narrative, to provide reliable, detailed, and specific causes of stillbirths and child deaths [23–25]. With the support of the Bill & Melinda Gates Foundation, CHAMPS was launched in 2017 in several high child-mortality countries to provide reliable data on cause-specific mortality. Accurate data on causes of death are fundamental to evidence-based health policy and public health interventions [26].

CHAMPS uses thorough postmortem diagnostic testing along with review of clinical records and some parts of the VA: the open narrative and raw answers to VA questionnaire, but not the VA-derived diagnoses. A local panel of experts reviews the information and assigns underlying, intermediate, and immediate causes of death, a process called Determination of Cause of Death (DeCoDe). This enables CHAMPS to produce high quality data on causes of death among under-five population using postmortem diagnostic techniques of MITS [27].

In this study, we compared the type, quality, and amount of CoD information generated from “VA only” and the CHAMPS method. We also assessed the concordance of the results generated from the two approaches. While CHAMPS generates specific microbiology and pathology diagnoses, this study focuses on the accuracy of the CoDs assigned by both systems. Methodologically speaking, although VA data was taken using the WHO 2016 questionnaire, the ICD-10 diagnosis determined by the CHAMPS methods was mapped to syndromic categories from the 2016 WHO Verbal Autopsy guideline for comparative purposes. With novel data from seven countries in Sub-Saharan Africa and Southeast Asia (Bangladesh, Ethiopia, Kenya, Mali, Mozambique, Sierra Leone, and South Africa), we aim to inform public health leaders and policymakers of the strengths and weaknesses, relevance, and consistency of these methods for identifying child mortality.

## Methods

### Study settings and design

The CHAMPS Network longitudinally collects robust and standardized data to understand and track preventable causes of childhood death in high-mortality areas. The CHAMPS network details have been published elsewhere [28–30]. All CHAMPS network sites are in research centers with pre-existing Demographic Health Surveillance Systems (HDSS) or have built capacity to closely follow up their catchment’s population, enabling them to conduct mortality surveillance. An HDSS is an open, dynamic cohort consisting of residents of a geographically defined area over time. The surveillance system tracks the occurrence of births, deaths, marriages, pregnancies, and migrations by enumerating these during routine household visits. To identify stillbirths and deaths of children under five as soon as they occur, mortality surveillance in CHAMPS sites also involves community informants, healthcare workers, and links with health facilities.

### Ethical statement

Ethical clearances from the respective institutions and national ethical clearance bodies have been secured for HDSS and CHAMPS activities. HDSS activities have standing approvals for continuing routine activities, including VA. All participants provided informed, voluntary, written consent. Consent was obtained from the responsible person in the family (the head of the household, the mother of the deceased child, or any eligible family member). To keep anonymity and confidentiality, we did not share data that contained participants’ personal identifiers with any third party.

### Data collection

CHAMPS study procedures have been published elsewhere [31]. Briefly, data from deaths identified through HDSS and mortality surveillance are collected prospectively from notified deaths in the communities and health facilities within the catchment. An <5 death or stillbirth identified within 24 hours, or 72 hours if refrigerated, whose family had been living in the catchment area for at least four to six months, is eligible to be enrolled for CHAMPS and requested to provide consent for Minimally Invasive Tissue Sampling (MITS). MITS includes postmortem collection of swabs, postmortem biopsies of vital organs, and body fluids for histopathologic and microbiologic examination. Clinical information found at the health facilities and the community where the stillbirths or death occurred is also collected, and families of the deceased are interviewed using the VA questionnaire, as described below.

### Verbal autopsy questionnaire

CHAMPS uses the WHO-2016 VA questionnaire, customized to include content enhancements, skip logic, and unit of measurement corrections for the CHAMPS study [32]. Questionnaires were translated into local languages and include information on age, sex, place of death, and symptoms observed during the late-life period of the deceased. The questionnaire also contains the symptom duration checklist, which is arranged loosely around anatomical systems and is intended to be informative for diagnosis of probable CoDs and narrowing the number of possible differential diagnoses.

### Causes of death assignment from VA

We used the InterVA-4 package from Open-VA to auto-generate the cause for each enrolled death [33, 34]. Open-VA uses Bayesian probabilistic modeling to assign likelihoods to causes of death based on coded responses to verbal autopsy questionnaires and ascribes corresponding ICD-10 codes [35, 36]; InterVA-4 algorithms do not consider information in the narrative section of the VA. This system mainly generates one likely CoDs and, if a single cause is not clear, three causes with probability values. The generated CoD with the highest probability was considered the underlying cause for comparison with the CoD assigned by CHAMPS DeCoDe.

### Determination of Cause-of-Death (DeCoDe) using minimally invasive tissue sampling (MITS)

Following the World Health Organization (WHO) application of the International Classification of Diseases–Version 10 (ICD-10), the DeCoDe expert panel determined the underlying cause and, for some deaths, one or more intermediate causes and an immediate CoD [35, 37]. We also compared the immediate CoDs assigned by the DeCoDe with the InterVA4’s underlying CoDs, as the InterVA4 does not designate immediate CoDs as the DeCoDe and found no significant difference; thus we decided Only to use the underlying CoDs assigned by DeCoDe for comparison purposes.

DeCoDe panels across the CHAMPS network follow a standard operating procedure and CHAMPS Diagnosis Standards [38].

The assigned causes of death by the DeCoDe panel were converted and categorized to the corresponding VA diagnosis using the 2016 WHO VA category definitions of the verbal autopsy standard [39]. The standard has a conversion table that shows and defines the VA diagnosis category and title with its corresponding ICD-10 codes. This conversion and categorization enable comparison of the generated CoD InterVA4 with the DeCoDe, which is considered a gold standard for concordance and accuracy.

### Quality control

Data collection was conducted by trained interviewers with least a high school education. They received a two-week training on the HDSS and VA questionnaires, recording, contacting close relatives, and data collection procedures. The training included sessions on discussing individual symptoms and their description in the local language for easy recognition by the respondents and demonstration of interviewing techniques by research team members. The field coordinators and supervisors continuously monitor data collection in the field to check progress and resolve problems that enumerators may have encountered during fieldwork.

### Inclusivity in global research

The study observed ethical, cultural, and scientific considerations specific to global research inclusivity, which is found in the **(S1 Checklist)**.

### Data management and analysis

Data were analysed using STATA version 16. Means and standard deviations (SDs) were presented for continuous variables, medians and interquartile ranges for skewed variables, and counts and percentages for categorical variables. Demographic characteristics included age, gender, occupation, religion, and household size. Variables with more than 45% missing data were excluded.

We considered stillbirth as the absence of life or spontaneous breathing after the viability of pregnancy (≥28 weeks of gestation) and before and during delivery. In addition, as most of the enrolled mothers who had stillbirths in each respective site did not remember or know their last normal menstrual period and were from rural areas, we took the death classification of stillbirths from the clinical records. Neonatal death was defined as a death in a live-born baby in the first 28 days of life. We classified neonatal death into very early, early, and late neonatal death if the death occurred in the first 24 hours (day 0), 1–6 days, and 7 to 28 days, respectively [40]. Infant death was defined as a baby’s death after 28 days of life and before the first birthday, and child death as death from the first birthday to before celebrating his/her 5^th^ year birthday [41].

Cause-specific mortality fractions (CSMF) for each surveillance site and CoD were computed by dividing the number of deaths due to specific causes assigned by either InterVA-4 and CHAMPS’s DeCoDe over the total number of deaths evaluated. The underlying causes of death from InterVA and DeCoDe were compared for agreement and pattern in assigning the diagnosis.

After the respective underlying causes of death that DeCoDe assigned were mapped and matched to its corresponding verbal autopsy standard, the agreement of both methods was evaluated using their concordance and accuracy of CSMF. We compared the CSMF of InterVA4 against DeCoDe using Lin’s Concordance Correlation Coefficient (LCC), [42] which was calculated using a user-defined command made for Stata–“Concord” [43].

The LCC determines how far the observed data deviate from the line of perfect concordance, a line at 45 degrees in a scatterplot. Lin’s coefficient increases in value as a function of the nearness of the data’s reduced major axis to the line of perfect concordance (the accuracy of the data) and of the tightness of the data about its reduced major axis (the precision of the data). The bias correction factor shows how far the best-line of shift is from the perfect concordance. The program (“Concord”) produces the LCC by multiplying the “Pearson correlation coefficient, r” with the bias-correction factor. Whereas the “Pearson correlation coefficient, r” is the measure of precision, the bias-correction factor is for accuracy [43].

The LCC was stratified across surveillance sites, age classification, and enrolment location to evaluate the performance of InterVA4. The stratification of the group was according to the WHO 2016 VA instrument guideline [36]: children aged < 1 year and aged 1–4 years. Accuracy is the measurement of the validity of a measurement’s exact value or how close the predicted value obtained in data is to the true value. Precision is defined as the degree of reproducibility of using the same measurement or procedure to measure the degree of consistency of independent measurements of the same variable [44]. The interpretation of the LCC we used is < 0.8 is poor, 0.81–90 –as good, and > 0.9 is excellent [45]. We also used the same interpretation for accuracy and precision.

Furthermore, to complement LCC in measuring the agreement between InterVA4 and DeCoDe, a mortality fraction ratio was calculated by dividing the CSMF generated by the InterVA4 with the DeCoDe’s (InterVA4 CSMF/DeCoDe CSMF) by surveillance site and for specific CoDs at a 95% confidence interval generated using the Koopman method to identify whether the interpretation between the two methods was lower or higher than expected [46]. This statistical method produces “the Koopman asymptotic score interval” for the ratio of probabilities in two-by-two contingency tables and works well for small sample sizes. The purpose of calculating these CIs was not to demonstrate statistical significance but to identify whether the CSMF ratio between InterVA–4 and DeCoDe interpretations was significantly lower or higher than that expected from chance, considering the number of cases involved.

## Results

**Fig 1:**
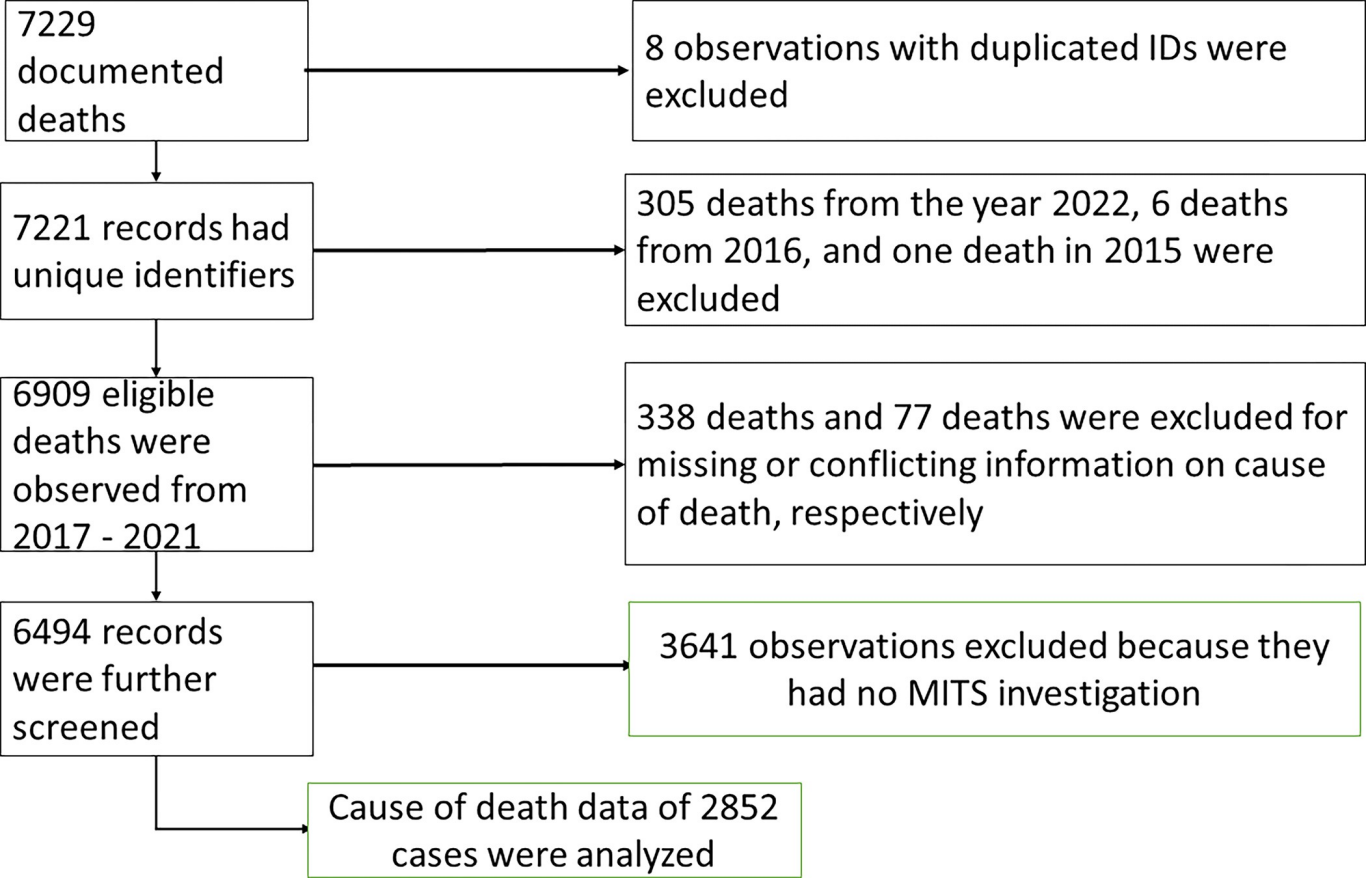
Flowchart of deaths included in the analysis.

CHAMPS sites identified 7221 unique deaths (including stillbirths), of which 6,909 (95.7%) were enrolled from February 1, 2017, through December 30, 2021 (**Fig 1**). Of 6,909 enrolled deaths, 338 (4.9%) observations were removed from the analysis because they were missing CoDs generated from the InterVA-4 package of the Open-VA because of transcription errors, and 77 were removed because of a conflicting date of birth or death and CoDs. These deaths were also removed from the analysis. Of the remaining 6494 deaths, 2340 (36.0%) were stillbirths, 2321 (35.7%) were neonates, 967 (14.9%) were infants, and 866 (13.3%) were children aged 1-<5 years. Of these, 3641 deaths were excluded as they were not enrolled for MITS and only had InterVA-generated CoD. Therefore, we analyzed 2853 (43.9%) of 6494 deaths enrolled for MITS and subsequently had CoD information generated from both DeCoDe and VA.

Of 2853 eligible deaths where both MITS and VA were undertaken, 1075 (37.7%) were stillbirths, 1077 (37.8%) neonatal deaths, 365 (12.8%) infant deaths, and 336 (11.9%) deaths of children aged 1–4 years. Around 30% (654) were enrolled from South Africa, 19% (545) from Kenya, 16.7% (476) from Mozambique, 12.2% (348) from Bangladesh, 11% from Sierra Leone (316) and Ethiopia (311), and 7% (203) from Mali. Across sites, the DeCoDe panel could not determine the CoDs for 78 (2.7%) of all deaths enrolled; of these, about half (40, 51.3%) were stillbirths, 15 (19.2%) were neonatal deaths, 15 (19.2%) infants, and 8 (10.3%) children aged 1–4 deaths (Table 1).

**Table 1:** Sociodemographic and mortality characteristics of deceased children under five years of age in seven countries with Child Health and Mortality Prevention Surveillance (CHAMPS) surveillance sites, overall and by site of enrollment.

Characteristics	Number (%) of deaths by CHAMPS site							Total (%)
	Bangladesh (%)	Ethiopia (%)	Kenya (%)	Mali (%)	Mozambique (%)	Sierra Leone (%)	South Africa (%)	
	N = 348	N = 311	N = 545	N = 203	N = 476	N = 316	N = 654	2853
Sex/gender								
Female	43.4	45.7	46.1	49.7	41.9	44.9	41.4	44.1
Male	56.6	54.3	53.9	50.3	58.1	55.1	58.6	55.9
Place of death								
Facility	97.1	92.6	76.7	78.3	90.8	91.1	94.3	89.0
Community	2.9	7.4	23.3	11.7	9.2	8.9	5.7	11.0
Season at time of death								
Dry season	70.4	67.8	50.9	59.1	40.8	54.4	53.8	55.1
Wet season	29.6	32.2	49.1	40.9	59.2	45.6	46.2	44.9
Mean gestational age for stillbirths in week*s	34.6 ^a^	-	34.0 ^b^	-	37.1 ^c^	-	32.8 ^d^	34.3 ^e^
Mean age at death in days for neonates	2.2	2.7	3.1	4.3	3.1	4.2	5.0	4.0
Mean age at death in months for infants	1.2	2.4	6.4	5.6	5.8	6.0	4.4	5.4
Mean age at death in months for children aged 1–4 years	17.4	26.0	28.7	29.0	27.0	25.3	27.4	27.2
Age group								
Stillbirth	50.9	64.0	29.4	39.4	43.5	31.3	23.4	37.7
Neonate (1–28 days)	48.0	28.6	29.4	36.0	34.9	27.5	51.2	37.8
Infant (29 days-11 months)	0.9	3.9	22.9	13.8	8.8	14.6	16.7	12.7
Child (1–4 years old)	0.3	3.5	18.3	10.8	12.8	26.6	8.7	11.8
Neonatal death timing (N = 1077) ^a^								
Very early (<24 hours)	50.9	51.7	58.1	37.0	49.4	27.6	32.5	43.3
Early (1–6 days)	40.1	38.2	25.6	38.4	37.9	52.9	39.7	38.2
Late (7–28 days)	9.0	10.1	16.3	24.6	12.7	19.5	27.8	18.5

*(%) = number of observations/column total (column percentage)*. *SD = standard deviation*. * Gestational age at birth was missing (unknown) for 949 observations. ^five^ observations, ^b^ one observation, ^c^ 40 observations, ^d^ 80 observations, ^e^ 126 observations

### Characteristics of study population and mortality groups across sites

Across sites, the mean age at death for U5 and newborns was 4 ± 10 months; females accounted for 44.1% of deaths (1251/2853), and more than half (55.1%) occurred in the dry season. The mean age of death was 4 (± 5.4) days for neonates, 5.4 (± 4) months for infants, and 2.2 (± 1.1) years. The mean gestational age for stillbirths was 34.3 weeks (95% CI 33.3, 35.6 weeks). Of 1075 recorded stillbirths, (207 (19.3%) were in Mozambique, 199 (18.5%) in Ethiopia, 177 (16.5%) in Bangladesh, 160 (14.9%) in Kenya, 153 (14.2%) in South Africa, 99 (9.2%) in Sierra Leone, and 80 (7.4%) in Mali. Overall, a large majority (89%) of deaths occurred in health facilities. About one-third of infant (111/365, 30.4%) and child deaths (102/336, 30.4%) occurred in the community. However, nearly all enrolled stillbirths (1037/1075, 96.5%) and neonatal deaths (1015/1077, 92.4%) occurred in health facilities (**Tables 1 and 2**).

**Table 2:** Correlation coefficients with 95% confidence interval for concordance in causes of death determined by InterVA4 compared to DeCoDe using MITS, among seven surveillance sites.

Number of Deaths		Concordance		Accuracy	Precision
		Correlation coefficient*	95% CI		
Surveillance Site					
Bangladesh	348	0.65	0.59–0.70	0.69	0.93
Ethiopia	311	0.83	0.79–0.86	0.83	0.99
Kenya	545	0.72	0.69–0.76	0.75	0.96
Mali	202	0.64	0.57–0.70	0.72	0.89
Mozambique	476	0.84	0.81–0.87	0.84	0.99
Siera Leone	316	0.72	0.67–0.78	0.74	0.98
South Africa	654	0.67	0.63–0.71	0.68	0.99
Overall	2852	0.75	0.73–0.76	0.76	0.98
Enrollment location					
Health facility	2540	0.76	0.74–0.78	0.77	0.98
Community	312	0.56	0.49–0.63	0.58	0.96
Sex					
Female	1256	0.75	0.73–0.78	0.77	0.98
Male	1595	0.74	0.72–0.76	0.75	0.99
Age group by site					
Age <1 year	2516	0.69	0.65–0.71	0.70	0.99
Bangladesh	347	0.47	0.44–0.55	0.53	0.89
Ethiopia	300	0.70	0.64–0.75	0.70	0.99
Kenya	445	0.73	0.69–0.77	0.74	0.98
Mali	180	0.58	0.49–0.66	0.64	0.90
Mozambique	415	0.76	0.72–0.80	0.77	0.99
Sierra Leone	232	0.72	0.66–0.77	0.75	0.96
South Africa	597	0.62	0.57–0.67	0.62	1.0
Children aged 1–4 years	366	0.28	0.19–0.37	0.30	0.91
Bangladesh	1	-	-	-	-
Ethiopia	11	0.18	0.00–0.61	0.22	0.81
Kenya	100	0.22	0.07–0.37	0.28	0.79
Mali	22	0.18	0.01–0.42	0.28	0.62
Mozambique	61	0.52	0.33–0.70	0.53	0.98
Siera Leone	84	0.17	0.01–0.36	0.17	0.98
South Africa	57	0.17	0.00–0.38	0.19	0.88

*Concordance Correlation Coefficient according to a method of Lin (Cox N, Steichen T. CONCORD: Stata module for concordance correlation. Statistical Software Components: Boston College Department of Economics; 2007 Retrieved from: https://ideas.repec.org/c/boc/bocode/s404501.html.). DeCoDe = Determination of Causes of Death by experts using Post-mortem Minimally Invasive Tissue Sampling

Precision is calculated by the “Concord” user-defined command and is correlated to Pearson’s coefficient.

The overall concordance of diagnoses across the surveillance sites and age groups was 0.75 (Table 2). The interVA4 method of assigning CoDs had better precision, but its accuracy compared to the DeCoDe was poor (<0.8). Stratified by surveillance sites, the overall concordance of all <5s deaths was lowest in Mali (0.64), and Ethiopia (0.83) and Mozambique sites (0.84) had good overall concordance.

**Fig 2:**
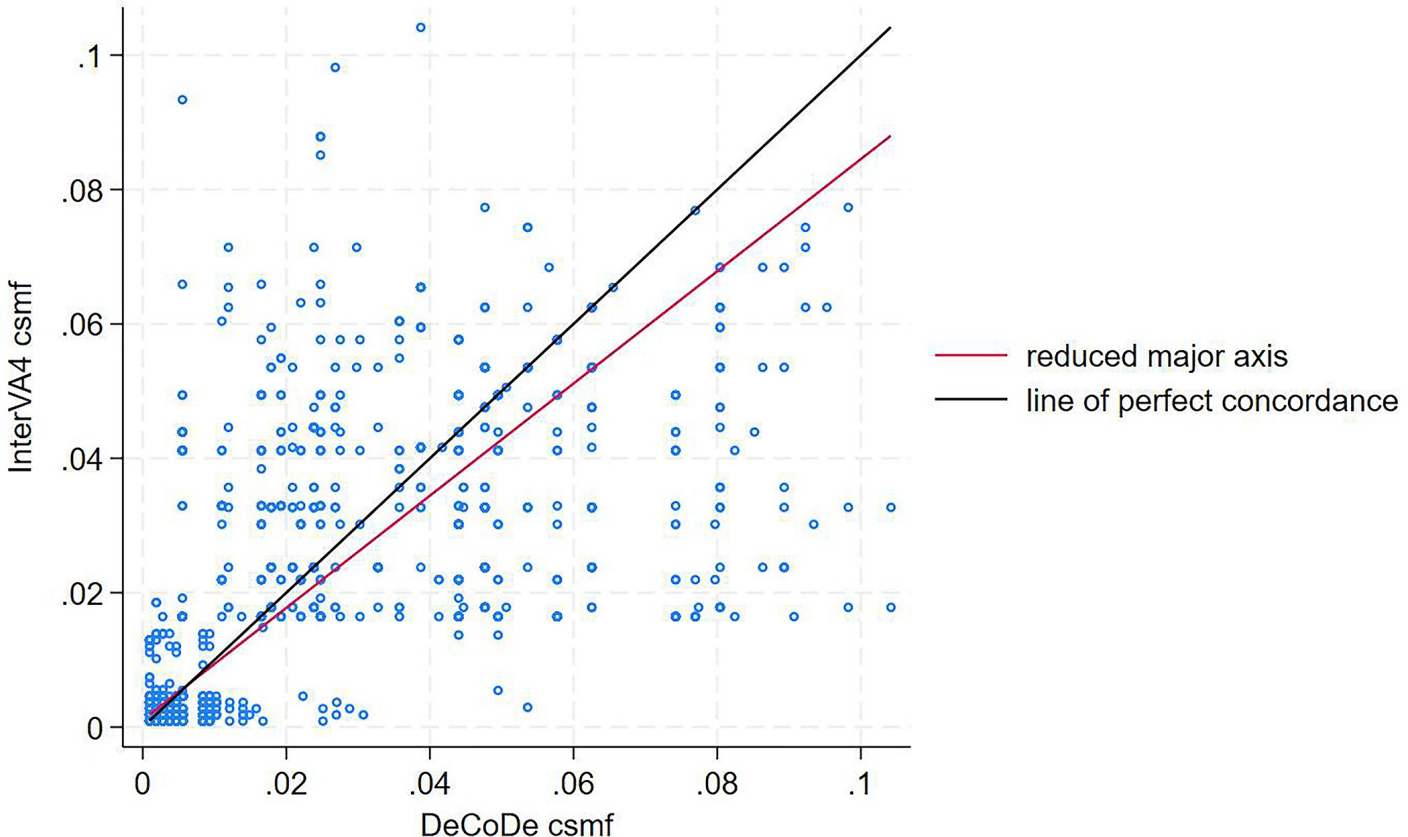
Concordance of cause-specific mortality fractions of the underlying causes of death between InterVA4 and DeCoDe for CHAMPS surveillance sites.

**Fig 3:**
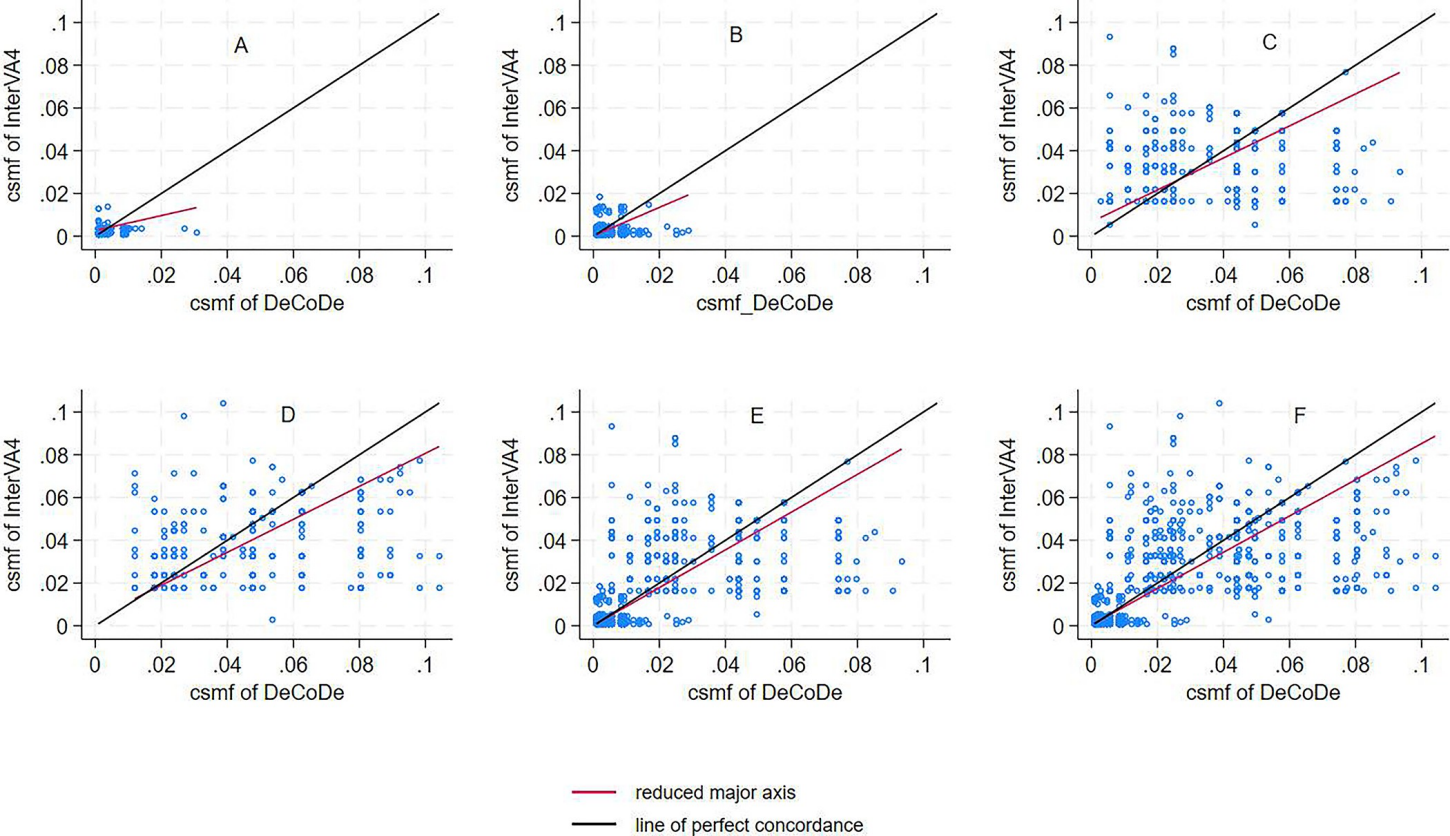
Concordance of cause-specific mortality fractions of the underlying causes of death between InterVA4 and DeCoDe for CHAMPS surveillance sites by age group.

The overall LCC of the CSMF generated by the InterVA4 against DeCoDe’s underlying causes of the death is poor, 0.75 (95%CI 0.73–0.76) (**Fig 2)**. The precision of the concordance was 0.98, while the accuracy was 0.76. The concordance coefficients were nearly the same across sexes for all CoDs and were higher for <5s enrolled at health facilities than those in the community. The determined CoDs for children aged < 1 year (0.69) were higher than those aged 1–4 years (0.28) despite their nearly no agreement when further stratified as stillbirths, neonates, and infants. However, the agreement considerably increased when those groups were combined **(Fig 3)**.

Cause-specific mortality fractions determined by InterVA4 and DeCoDe differed in important ways for some of the more common diseases (Table 3). In those surveillance sites where the DeCoDe panels determined HIV as the underlying CoD for some deaths, the InterVA4 model predicted considerably fewer HIV deaths, as demonstrated by CSMF. This pattern is also seen in many sites for diagnoses such as malnutrition (Ethiopia, Kenya, Mali, and Sierra Leone), neonatal sepsis (all sites except Mali), and birth asphyxia (all sites). However, the InterVA4 predicted a substantially higher proportion of deaths caused in most sites by prematurity (all sites, except Mali), malaria (Kenya, Mali, Mozambique, and Sierra Leone), malnutrition (Mozambique and South Africa), diarrheal diseases (except in Mozambique, which was lower), and meningitis (Kenya, Mozambique, Sierra Leone, and South Africa) than did DeCoDe.

**Table 3:** Cause-specific mortality fractions of the underlying causes of deaths of under-five deaths among seven surveillance sites by InterVA4 and DeCoDe methods and sorted by WHO 2022 verbal autopsy categories.

Causes of death	Cause-specific mortality fractions, in percent, by CHAMPS site and cause attribution method													
	Bangladesh (348 deaths)		Ethiopia (311 deaths)		Kenya (545 deaths)		Mali (202 deaths)		Mozambique (476 deaths)		Sierra Leone (316 deaths)		South Africa (645 deaths)	
	Inter VA4*	DeCoDe	InterVA4	DeCoDe	InterVA4	DeCoDe	InterVA4	DeCoDe	InterVA4	DeCoDe	InterVA4	DeCoDe	Inter VA4	DeCoDe
01.01 Sepsis (non-obstetric)	-	-	0.96	0.64	0.18	1.10	-	1.49	0.42	0.63	-	3.80	-	0.61
01.02 Acute resp. Infect, incl. pneumonia	-	0.29	0.64	1.61	6.61	4.04	5.45	2.97	3.36	3.99	8.54	3.48	8.87	2.91
01.03 HIV/AIDS	-	-	0.32	-	2.94	5.14	0.99	2.48	2.73	4.20	0.95	2.22	0.92	1.99
01.04 Diarrheal diseases	-	-	1.29	0.32	5.14	2.20	1.49	-	4.62	5.04	4.75	0.95	2.29	1.68
01.05 Malaria	0.29	-	0.32	-	8.99	7.16	7.92	1.49	3.57	2.31	15.51	9.81	1.07	-
01.06 Measles	-	0.29	0.32	0.32	-	-	-	0.5	-	-	-	-	-	-
01.07 Meningitis and encephalitis	0.57	-	0.96	-	5.69	0.18	1.49	-	0.84	0.42	2.85	0.63	2.29	1.07
01.09 Pulmonary tuberculosis	-	-	-	-	-	-	-	-	-	0.21	-	-	-	-
01.10 Pertussis	-	-	-	-	-	0.18	0.50	0.50	-	0.42	-	0.32	-	-
01.12 Dengue fever	-	-	-	-	0.18	-	-	-	0.21	-	-	-	0.15	-
01.99 Other and unspecified infect dis.	-	-	1.29	-	5.69	0.37	1.98	0.50	2.10	1.47	5.38	0.95	2.45	1.68
02.02 Digestive neoplasms	-	-	-	-	-	-	-	-	-	-	-	-	-	0.15
02.99 Other and unspecified neoplasms	-	-	-	-	-	0.37	-	0.5	-	-	-	-	-	0.61
03.01 Severe anemia	-	-	-	-	-	1.10	-	0.99	-	-	-	0.32	-	-
03.02 Severe malnutrition	0.29	0.29	0.96	5.14	2.20	10.28	0.50	2.97	2.10	0.84	0.32	14.56	1.53	0.76
03.03 Diabetes mellitus	-	-	-	-	0.18	0.18	-	-	-	-	-	-	0.15	-
04.02 Stroke	-	-	-	-	0.18	-	-	-	1.05	-	-	-	0.15	-
04.99 Other and unspecified cardiac dis.	-	-	-	-	-	-	-	0.50	0.21	-	-	-	-	0.61
05.02 Asthma	-	-	-	-	-	-	-	-	-	-	-	-	-	0.31
06.01 Acute abdomen	-	-	-	-	0.18	-	-	0.50	-	-	0.32	-	0.76	-
06.02 Liver cirrhosis	-	-	-	-	-	0.18	-	0.50	-	-	-	-	0.15	0.46
07.01 Renal failure	-	-	-	-	-	-	-	-	-	-	-	-	0.15	-
08.01 Epilepsy	-	-	0.64	-	2.57	-	2.97	-	0.42	0.21	0.95	-	2.45	0.31
10.01 Prematurity	29.89	-	13.18	9.97	17.25	4.04	8.42	13.37	35.29	1.26	12.66	3.80	38.99	31.50
10.02 Birth asphyxia	12.93	66.67	16.72	48.87	12.29	42.39	12.38	32.67	11.34	56.93	9.81	40.51	9.02	20.34
10.03 Neonatal pneumonia	0.86	-	2.57	2.25	2.57	2.39	4.95	1.49	1.05	5.67	4.11	2.53	1.83	2.45
10.04 Neonatal sepsis	1.15	3.45	1.61	4.50	2.02	5.69	8.42	3.47	1.05	4.62	3.48	4.75	1.53	8.87
10.06 Congenital anomaly	-	3.45	-	18.65	-	3.12	-	14.85	-	5.25	-	2.85	-	11.47
10.99 Other and unspecified neonatal CoD	0.29	3.74	-	2.25	-	2.39	2.48	5.94	-	3.15	-	0.63	0.15	4.74
11.01/11.02 Fresh and macerated stillbirths	51.45	0.57	58.20	5.14	23.67	2.39	39.11	5.45	28.57	1.68	28.80	6.65	21.25	1.83
12.01 Road traffic accident	2.30	-	-	-	-	-	0.50	-	0.42	-	0.32	-	0.46	0.15
12.02 Other transport accident	-	-	-	-	0.73	-	0.50	-		-	0.32	-	1.22	-
12.03 Accid. fall	0.29	-	-	-	0.37	-	-	-	0.42	-	0.63	-	0.61	-
12.05 Accid. Expos to smoke, fire, flame	-	-	-	-	0.18	-	-	-	0.21	-	0.32	-	0.76	-
12.07 Accid. Poisoning and noxious subs					0.18	0.18	-	-	-	-	-	-	0.15	-
12.09 Assault	-	-	-	-	-	0.18	-	-	-	-	-	-	0.31	-
12.10 Exposure to a force of nature	-	-	-	-	-	-	-	-	-	-	-	-	0.15	-
12.99 Other and unspecified external CoD	-	-	-	0.32	-	1.28	-	1.98	-	0.21	-	0.95	0.15	3.21
98 Other and unspecified NCD	-	-	-	-	-	3.49	-	4.95	-	1.47	-	0.32	-	2.29

CSMF = Cause Specific Mortality Fraction. DeCoDe methods = Experts Determination of Cause of Death by experts using post-mortum Minimally invasive Tissue Sampling. CoD = Cause of Death. CSMF ratio = CSMF InterVA4 divided by CSMF of DeCoDe. NCD = Non-communicable Disease. Dis = disease. Accid. = accidental. 95% CI = 95% Confidence Interval

Some CoDs were exclusively assigned by the InterVA4 model rather than the postmortem DeCoDe. For example, acute abdomen, renal failure, dengue fever, stroke, road and other traffic accidents, accidental falls, and exposure to the force of nature were exclusively assigned by the interVA4 model as the underlying CoDs and were not determined by the experts using postmortem MITS. Conversely, unspecified external causes of death, unspecified non-communicable diseases, congenital anomalies, digestive neoplasms, and unspecified neoplasms were exclusively determined as CoDs by experts using postmortem MITS. In addition, only the DeCoDe panels ascertained pulmonary tuberculosis as the CoD in South Africa. At the same time, the InterVA4 model did not predict it. The InterVA4 model exclusively assigned epilepsy in Ethiopia, Kenya, Mali, and Sierra Leone, but both InterVA4 and DeCoDe noted epilepsy as an underlying CoD in Mozambique and South Africa.

The InterVA4 CSMF and DeCoDe CSMF ratios were calculated with a 95% confidence interval using the CSMFs tabulated in Table 3 to show that many of these differences did not occur by chance. The InterVA4 CoDs of fresh and macerated stillbirths had the highest CSMF ratio, and unspecified neonatal CoD had the lowest CSMF ratio.

## Discussion

This study compared the InterVA4 model with experts’ determination of CoDs using advanced diagnostics and postmortem MITS. It showed poor InterVA4 agreement and concordance in predicting the causes of death against DeCoDe among our <5s studied deaths. The concordance suffered from its accuracy (< 0.8), although the precision was good (>0.8).

Several other studies compared the InterVA4 with Physicians-Certified Verbal Autopsy (PCVA) and other standardized verbal autopsy diagnoses for public health equivalence to test its functionality and costs [16, 47–50]. Others have also studied the performance of InterVA4 with postmortem histologic findings. Knowing whether these tools lead to similar conclusions—and if not, how results differ—is important before relying on verbal autopsy-generated information as the general country-wide source of CoD and for planning and executing public health interventions [13]. This concept is particularly crucial in a setting without widespread mortality registrations and in resource-constrained areas.

Across surveillance sites, there were considerable differences in the two systems’ concordance, as Ethiopia and Mozambique’s LCC were good (>0.8) while the others were poor. Our findings could be explained by quality differences in collecting the VA data and the extent of CHAMPS’s concurrent utilization of VA data with other clinical information to assign the CoDs. The considerable agreement differences between deaths identified in health institutions and the community also substantiate the argument, as death enrolled in the health facility would have rich clinical information besides the VA, compared to those cases enrolled in the community. Furthermore, other studies have reported that the extent and way of VA data collection determined how the InterVA4 assigned the respective CoDs [10, 16, 47, 51, 52].

However, we found the overall agreement in assigning the CoDs between the two systems to be poor. This finding is unsurprising as several studies also found significant differences between InterVA4 and PCVA or histologic findings [48, 53]. The concordance of InterVA4 considerably decreases for stillbirths, neonates, and infants at individual and population levels [33]. However, when they are combined, the level of agreement improves significantly. Most importantly, more than a quarter of the overall sample were stillbirths, where the InterVA4 is not designed to predict the causes of death. For example, most of the diagnoses assigned by the InterVA4 for stillbirths were VAs-11.01 or VAs-11.02. These assigned “macerated or fresh stillbirths” corresponded to the ICD-10 code of P95. In addition, InterVA4 did not assign congenital anomalies arising during the prenatal period, limiting its CoD equivalence compared to DeCoDe’s.

Our findings did not agree with other studies that indicated an excellent concordance between the assigned causes of death between the InterVA4 model and several PCVA findings [47]. InterVA4 performed well in identifying malnutrition and certain perinatal conditions as the underlying CoDs, similarly to the DeCoDe. For example, the ratio of the proportion of malnutrition, birth asphyxia, and prematurity was closer to one or slightly higher, meaning strong equivalence in assigning those conditions.

The DeCoDe captures the overall mortality chain from underlying, intermediate, and immediate causes of death, which is not done with InterVA4. In this study, we could only compare the InterVA4 models’ most likely underlying causes of death to the underlying causes attributed to DeCoDe. Comparing only the underlying CoDs may potentially limit the overall correlation of causes of death between the two approaches, as many deaths in live-born children occur after a complicated course of multiple causes [54]. For example, a neonate born prematurely could die of sepsis after admission to an intensive care unit; in this case, DeCoDe would account for both causes. The InterVA4, however, would mostly likely predict either of the causal chains, missing the overall causal chain. These complete causal-chain scenarios identified by the DeCoDe panel would be based on pieces of evidence from MITS and microbiological, clinical, and VA data. The DeCoDe process does involve clinical judgment in some cases, as attributing causes of death from multiple results can be complex, and clinical information, in particular, can be incomplete, incorrect, or absent [55]. Nonetheless, errors should be few as the procedure is designed to use the best possible set of information.

Another difference between the two methods is that the InterVA4 model mostly tends to assign the stillbirths–either fresh or macerated—underlying CoDs in 80% of the cases, while the remaining CoDs designated were prematurity and intrauterine hypoxia; these CoDs, that the InterVA4 mainly assigned for stillbirths were VA-11.01 and VA-11.02 corresponds to the ICD code P95 –undetermined or unspecified causes of death. There were substantial differences in assigning ICD code P95 between the two methods unrelated to chance, as the InterVA4 assigned more than the DeCoDe. The differences could arise from the VA data quality, the algorithm design, or the MITS’s accuracy in determining the most likely causes of death.

Similarly, prematurity was also more often assigned by InterVA4 than the DeCoDe across sites, which could also be related to the level of certainty in determining the pregnancy’s gestational age, which the DeCoDe panel uses when assigning prenatal mortality. Moreover, those babies born prematurely are most likely to have impending birth asphyxia or respiratory distress, and the DeCoDe panel assigned more birth asphyxia than the InterVA4 model. These differences point out the relationship and the complexities of the causal chain that were responsible for the <5s deaths.

Despite the richness of the data from these HDSS sites, our study had several limitations. While CHAMPS’ methods produce high-quality cause-of-death information for those children evaluated, the postmortem diagnostic testing protocols require rapid death identification and collection of specimens before a child is buried. Therefore, many deaths in CHAMPS catchment areas do not undergo such testing; in contrast, evaluation of deaths using VA can be done after burial, at a family’s convenience, which is usually 2 to 12 weeks [27]. Causes of death captured using automated verbal autopsy have inherent limitations, although methods continuously evolve. However, the processes used in verbal autopsy–sourcing information from available respondents who have varying knowledge and insights about the history, symptoms, and signs leading to someone else’s death–cannot be expected to provide specific causes for every death in a community. In addition, Open VA could not identify the underlying causes of death for stillbirths despite having significant limitations in ascertaining their death category using their gestational age.

Even though the Open VA program pragmatically derives the causes of death in terms of public health importance and feasibility using verbal autopsy at the population level, it does not capture other causes with more general symptoms like abdominal pain and malaria [36, 37, 49]. Lastly, Open VA, in modeling causes of death on a case-by-case basis, does not have any input characterizing the socioeconomic status of the deceased. HDSS data provide valid subnational estimates, but their representativeness of broader populations may vary.

## Conclusion

Our findings point out that VA diagnosis alone, as generated by InterVA4, often incorrectly predicts causes of death among <5s, using DeCoDe findings as the gold standard. The InterVA4 model lacks precision in determining the underlying causes of death and cannot predict some conditions like congenital anomalies. Future improvement of the reliability and validity of VA data by strengthening the quality of data collection and automatically assigning CoDs using robust and new technologies, such as artificial intelligence, is recommended. Improving models to better predict causes of death, perhaps by using information from deaths that also have information from postmortem diagnostic assessments such as DeCoDe, would improve the usefulness of VA as a tool to inform health policies [56, 57].

Overall, the role of the VA as a tool for diagnosing and tracking the progress of mortality data among U5s is essential despite the noted shortcomings. Using the DeCoDe process that combines Minimally invasive tissue sampling (MITS) and other techniques could provide data to help improve CoDs determination. The data should subsequently be utilized to improve the CoD determination algorithms of VA and its diagnostic ability.

## Supporting information

S1 Checklist Inclusivity in global health. (DOCX)
