# Correction: Comparison of causes of stillbirth and child deaths as determined by verbal autopsy and minimally invasive tissue sampling

**DOI:** 10.1371/journal.pgph.0005586

**Published:** 2025-12-05

**Authors:** Nega Assefa, J. Anthony G. Scott, Lola Madrid, Merga Dheresa, Gezahegn Mengesha, Shabir Madhi, Sana Mahtab, Ziyaad Dangor, Nellie Myburgh, Lesego Kamogelo Mothibi, Samba O. Sow, Karen L. Kotloff, Milagritos D. Tapia, Uma U. Onwuchekwa, Mahamane Djiteye, Rosauro Varo, Inacio Mandomando, Ariel Nhacolo, Charfudin Sacoor, Elisio Xerinda, Ikechukwu Ogbuanu, Solomon Samura, Babatunde Duduyemi, Alim Swarray-Deen, Abdulai Bah, Shams El Arifeen, Emily S. Gurley, Mohammed Zahid Hossain, Afruna Rahman, Atique Iqbal Chowdhury, Quique Bassat, Portia Mutevedzi, Solveig A. Cunningham, Dianna Blau, Cynthia G. Whitney

The second, sixth, 24th, 31st, and 35th authors’ names are spelled incorrectly. The correct names are: J. Anthony G. Scott, Shabir Madhi, Alim Swarray-Deen, Quique Bassat, and Cynthia G. Whitney. The correct citation is: Assefa N, Scott JAG, Madrid L, Dheresa M, Mengesha G, Madhi S, et al. (2024) Comparison of causes of stillbirth and child deaths as determined by verbal autopsy and minimally invasive tissue sampling. PLOS Glob Public Health 4(7): e0003065. https://doi.org/10.1371/journal.pgph.0003065.
